# Supplementary material for: Achieving symptom relief in patients with myalgic encephalomyelitis by targeting the neuro-immune interface and optimizing disease tolerance
Source: Oxf Open Immunol. 2023 Apr 17;4(1):iqad003. doi: 10.1093/oxfimm/iqad003 (PMC10148714; doi:10.1093/oxfimm/iqad003)
Supplement: iqad003_Supplementary_Data [file iqad003_supplementary_data.docx]

**Supplementary Materials:**

**Table S1. List of antibodies used in mass cytometry**

| **Metal Tag** | **Antigen** | **Clone** | **Vendor** |
| --- | --- | --- | --- |
| Y89 | CD45 | HI30 | Fluidigm |
| In113 | HLA-ABC | W6/32 | BioLegend |
| In115 | CD57 | HCD57 | BioLegend |
| La139 | TCR Vα7.2 | 3C10 | BioLegend |
| Nd142 | CD19 | HIB19 | Fluidigm |
| Nd143 | CD5 | UCHT2 | BioLegend |
| Nd144 | CD16 | 3G8 | BioLegend |
| Nd145 | CD4 | RPA-T4 | BioLegend |
| Nd146 | CD8a | SK1 | BioLegend |
| Sm147 | CD11c | Bu15 | Fluidigm |
| Nd148 | CD31 | WM59 | BioLegend |
| Sm149 | CD25 | 2A3 | Fluidigm |
| Nd150 | CD64 | 10.1 | Biolegend |
| Eu151 | CD123 | 6H6 | BioLegend |
| Sm152 | γδTCR | 5A6.E9 | Fischer S |
| Eu153 | CD13 | WM15 | Biolegend |
| Sm154 | CD3e | UCHT1 | Fluidigm |
| Gd155 | CD7 | CD7-6B7 | Biolegend |
| Gd156 | CD26 | BA5b | Biolegend |
| Gd157 | CD9 | SN4 C3-3A2 | eBio |
| Tb159 | CD22 | HIB22 | Biolegend |
| Gd160 | CD14 | M5E2 | BioLegend |
| Dy161 | CD161 | HP-3G10 | BioLegend |
| Dy162 | CD29 | TS2/16 | Biolegend |
| Dy163 | HLA-DR | L243 | BioLegend |
| Dy164 | CD44 | BJ18 | BioLegend |
| Ho165 | CD127 (IL-7Rα) | A019D5 | Fluidigm |
| Er166 | CD24 | ML5 | BioLegend |
| Er167 | CD27 | L128 | Fluidigm |
| Er168 | CD38 | HIT2 | BioLegend |
| Tm169 | CD45RA | HI100 | Fluidigm |
| Er170 | CD20 | 2H7 | BioLegend |
| Yb171 | CD33 | WM53 | Biolegend |
| Yb172 | IgD | IA6-2 | BioLegend |
| Yb173 | CD56 | HCD56 | BioLegend |
| Yb174 | CD99 | HCD99 | Biolegend |
| Lu175 | CD15 | W6D3 | Biolegend |
| Yb176 | CD39 | A1 | BioLegend |
| Ir191 | Cell-ID™ Intercalator-Ir (DNA) | NA | Fluidigm |
| Ir193 | Cell-ID™ Intercalator-Ir (DNA) | NA | Fluidigm |
| Bi209 | CD11b | Mac-1 | Fluidigm |
